# Hsa_circ_0062682 Promotes Serine Metabolism and Tumor Growth in Colorectal Cancer by Regulating the miR-940/PHGDH Axis

**DOI:** 10.3389/fcell.2021.770006

**Published:** 2021-12-08

**Authors:** Shengbai Sun, Chaoqun Li, Kaisa Cui, Bingxin Liu, Mingyue Zhou, Yulin Cao, Jia Zhang, Zehua Bian, Bojian Fei, Zhaohui Huang

**Affiliations:** ^1^ Wuxi Cancer Institute, Affiliated Hospital of Jiangnan University, Wuxi, China; ^2^ Laboratory of Cancer Epigenetics, Wuxi School of Medicine, Jiangnan University, Wuxi, China; ^3^ Department of Gastrointestinal Surgery, Affiliated Hospital of Jiangnan University, Wuxi, China

**Keywords:** circular RNA, colorectal cancer, miR-940, PHGDH, serine metabolism

## Abstract

Colorectal cancer (CRC) is one of the most common malignancies globally. Increasing evidence indicates that circular RNAs (circRNAs) play a pivotal role in various cancers. The present study focused on exploring the role of a functionally unknown circRNA, hsa_circ_0062682 (circ_0062682), in CRC. By online analyses and experimental validations, we showed that circ_0062682 expression was aberrantly increased in CRC tissues compared with paired normal tissues. Increased expression of circ_0062682 in CRC notably correlated with a poor prognosis and advanced tumor stage. Functional experiments showed that circ_0062682 knockdown reduced CRC growth both *in vitro* and *in vivo*. Mechanistically, we revealed that circ_0062682 could sponge miR-940 and identified D-3-phosphoglycerate dehydrogenase (PHGDH), a key oxidoreductase involved in serine biosynthesis, as a novel target of miR-940. Silencing miR-940 expression could mimic the inhibitory effect of circ_0062682 knockdown on CRC proliferation. The expression of PHGDH was downregulated in circ_0062682-depleted or miR-940 overexpressing CRC cells at both the mRNA and protein levels. Circ_0062682 knockdown suppressed CRC growth by decreasing PHGDH expression and serine production via miR-940. Taken together, these data demonstrate, for the first time, that circ_0062682 promotes serine metabolism and tumor growth in CRC by regulating the miR-940/PHGDH axis, suggesting circ_0062682 as a potential novel therapeutic target for CRC.

## Introduction

Colorectal cancer (CRC) is one of the most common malignancies, ranking third in terms of incidence and second in terms of mortality globally (Sung et al., 2021). The incidence of CRC is rising rapidly in developing countries and is over 4 times higher than that in developed countries (Sung et al., 2021). Due to metastasis and recurrence, the prognosis of CRC patients remains poor, and the underlying mechanisms for CRC are still poorly elucidated (Navarro et al., 2017).

Circular RNA (circRNA) is a new type of non-coding RNA (ncRNA) that forms a covalently closed loop without terminal 5′ caps and 3′ polyadenylated tails (Kristensen et al., 2019). Due to their closed circular structure, circRNAs are more stable and resistant to RNase R than linear transcripts, highlighting them as promising biomarkers (Chen, 2020). With the development of high-throughput RNA sequencing (RNA-seq) and circRNA-specific bioinformatics prediction, an increasing number of circRNAs have been identified (Meng et al., 2017). It is worth noting that circRNAs can act as miRNA sponges to regulate the expression of target genes (Ashwal-Fluss et al., 2014). CircRNAs are functionally involved in mediating various cellular processes, including cell proliferation, differentiation and metabolism (Patop et al., 2019; Yin et al., 2019). Disorders of circRNAs are also associated with a variety of human diseases, including cancers (Ma et al., 2020). However, the role of circRNAs in regulating the metabolic adaptation of cancer cells in a nutrient-limited microenvironment is unclear.

Cancer cells, including CRC cells, can shift their metabolic pathways in response to nutrient availability, while the underlying mechanisms remain largely elusive (Boroughs and DeBerardinis, 2015). Apart from enhancing glycolysis, many rapidly proliferating cells, including cancer cells, are also highly dependent on serine. Increased serine biosynthesis is considered an important metabolic change in cancer cells, which generates more serine from the glycolytic intermediate 3-phosphoglycerate (3-PG), contributing to the production of glutathione (GSH) and nucleotides (Amelio et al., 2014; Li and Ye, 2020; Pan et al., 2021). However, how altered serine metabolism affects cancer progression under metabolic stress is largely unknown.

In this study, we revealed that circ_0062682 was aberrantly overexpressed in CRC tissues and was associated with poor clinical outcomes. By a series of functional and mechanistic investigations, we revealed that circ_0062682 could promote serine metabolism and CRC growth by sponging miR-940 and increasing the expression of PHGDH, a novel target of miR-940. Our findings reveal a novel circ_0062682/miR-940/PHGDH axis that regulates serine metabolism and tumorigenesis, and highlight circ_0062682 as a potential biomarker and therapeutic target for CRC.

## Materials and Methods

### Data Resources and Tissue Collection

The microarray expression data of circRNAs were downloaded from the Gene Expression Omnibus (GEO). A total of 83 paired CRC and corresponding adjacent non-tumorous tissues (NCT) were collected from patients at the Affiliated Hospital of Jiangnan University with written informed consent. All collected tissues were immediately frozen with liquid nitrogen and then stored at −80°C until use. Our study (LS2019010) was approved by the Research Ethics Committee of Affiliated Hospital of Jiangnan University. The detailed patient information is described in Supplementary Table S1.

### Cell Culture

Human CRC cell lines (HCT8, DLD1, HCT116 and HT29), normal colorectal epithelial cell lines (NCM460 and CCC-HIE-2) and HEK-293T (293T) cells were purchased from the American Type Culture Collection (ATCC). All of these cell lines were cultured according to the instructions recommended by ATCC, and characterized by Genewiz (China) using short tandem repeat markers.

### Nucleic Acid Extraction and qRT-PCR

Nuclear and cytoplasmic fractions of CRC cells were separated using the PARISTM Kit (Thermo Fisher, United States). Total RNA was extracted from cells, tissues or subcellular fractions with the RNA isolater Total RNA Extraction Reagent (Vazyme, China), and was then reverse transcribed into complementary DNA (cDNA) using the HiFiScript cDNA Synthesis Kit (CWBIO, China). The expression levels of circ_0062682 and PHGDH were detected with UltraSYBR Mixture (CWBIO) using qRT-PCR on the SLAN PCR System (Hongshi Tech, China). The expression of miR-940 was detected using a stem-loop method as we previously described (Jin et al., 2019). Genomic DNA (gDNA) was extracted from CRC cells using the TIANamp Genomic DNA Kit (TIANGEN, China). All primers are listed in Supplementary Table S2.

### Vector Construction, Oligonucleotide Transfection and Stable Cell Line Construction

The siRNAs targeting circ_0062682, miR-940 mimics, miR-940 inhibitors and their negative control (NC) were synthesized by GenePharma (China). Validated shRNA sequences of circ_0062682 were synthesized and cloned into the pLKO.1 shRNA lentivector. The 3′UTRs of PHGDH and circ_0062682 were amplified from human gDNA using PhantaMaster Mix (Vazyme) and cloned into the luciferase reporter vector pLUC. Mutant 3′UTRs of PHGDH and circ_0062682 were constructed by overlap extension PCR and cloned into pLUC (Li M. et al., 2019). All plasmids and oligonucleotides were transfected into cells using Lipofectamine 2000 (Invitrogen, United States). Lentivirus plasmids were transfected into 293T cells along with the packaging plasmids using Lipofectamine 2000. Virus particles were harvested 48 h after transfection and then separately used to infect HCT8 or DLD1 cells to obtain corresponding stable cell lines. The related sequences are listed in Supplementary Table S2.

### RNase R and Actinomycin D Treatment

Total RNA was incubated with or without 2 U/μg RNase R (Geneseed, China) at 37°C for 30 min and subsequently at 70°C for 5 min to inactive the enzyme. A final concentration of 1 μg/ml actinomycin D was used to treat CRC cells (Thermo Fisher) for different times, and the stability of circ_0062682 and TPST2 in these treated cells was evaluated by qRT-PCR.

### CCK8 and Colony Formation Assays

Cell viability was detected using the Cell Counting 8 Kit (CCK8, Beyotime, China). For the colony formation assay, a total of 1000 CRC cells were seeded into 6-well plates and continually cultured for 10–14 days. The colonies were fixed with 4% paraformaldehyde and stained with 0.1% crystal violet. The number of clones was counted using an inverted microscope (Olympus, Japan).

### EdU and Immunofluorescence Assays

Cells were cultured in medium containing 10 μM EdU (Beyotime) for 2 h and then collected and fixed with 4% paraformaldehyde. After washing with PBS, cells were treated with 0.5% Triton X-100 for 10 min at room temperature, and were then incubated with 1 × click buffer at room temperature for 30 min in the dark. Cells were capped using fluorescent mounting medium with DAPI (ZSGB Bio, China), and captured using a fluorescence microscope (Olympus). For the immunofluorescence assay, CRC cells grown on coverslips were fixed with 4% paraformaldehyde and permeabilized with 0.5% Triton X-100. After blocking with 10% goat serum, the cells were incubated with Ki67 antibodies (1:200, Abclonal, China) at 4°C overnight. These samples were then incubated with the corresponding secondary antibodies and capped using fluorescent mounting medium with DAPI (ZSGB Bio). Images were captured by a fluorescence microscope (Olympus).

### Cell Cycle Analyses

CRC cells were synchronized by serum starvation for 24 h, and released into the cell cycle by adding serum. These cells were then harvested and fixed with ice-cold 70% ethanol. The fixed cells were subjected to cell cycle analyses using the Cell Cycle and Apoptosis Detection Kit (CWBIO).

### Luciferase Reporter Assay

HEK-293T and HCT8 cells were transiently transfected with the constructed pLUC plasmids and miRNA mimics with the Renilla luciferase plasmid as an adjusting control among different wells. Thirty-six hours after transfection, the cells were harvested and then assayed for luciferase activity with the Dual-Luciferase Reporter Assay System (Beyotime).

### Tumor Formation Assay in a Nude Mouse Model

CRC cells were subcutaneously injected into the same flank of athymic male BALB/c nude mice at 5 weeks of age (*n* = 8 for each group). Three weeks after injection, the mice were sacrificed and inspected for subcutaneous tumors growth (Bian et al., 2018). The animal handling procedures were performed in accordance with the National Institutes of Health’s Guide for the Care and Use of Laboratory Animals.

### Western Blotting

Cells were lysed in RIPA buffer supplemented with Cocktail (Roche, Switzerland). The concentrations of proteins were quantified using the BCA Protein Assay Kit (Vazyme) and then separated on 10% SDS-PAGE gels. When the electrophoresis was finished, proteins were transferred to a NC membrane (Millipore, United States). After being blocked in 5% skimmed milk powder for 1 h, the membranes were incubated with primary antibodies against PHGDH (1:1000, Proteintech) and GAPDH (1:5000, ABclonal) overnight at 4°C. A peroxidase-conjugated secondary antibody (1:5000, Thermo Fisher) was used to incubate the membranes for 1 h at room temperature, and the membranes were then visualized with ECL substrate (Vazyme) on a BioRad system (BioRad, United States).

### Cellular ROS Detection

Intracellular ROS levels were detected using the Reactive Oxygen Species Assay Kit (Beyotime) according to the manufacturer’s instructions. Cells were incubated with 10 μM H_2_DCF-DA at 37°C for 20 min and measured with a flow cytometry (BD, United States).

### Serine and Glycine Measurement

Cells were harvested with precooled 70% methanol and incubated at −80°C for 24 h. Sulfosalicylic acid (10%) was added to the lysate, mixed at a 1:1 volume, and stored at 4°C for 8 h. The lysate was centrifuged at 13,000 rpm for 10 min, and the supernatants were filtered and transferred to 1.5 ml liquid phase vials. The samples were then tested by a liquid chromatography-mass spectrometry assay.

### H&E and immunohistochemistry Staining

Tissue samples were fixed in 4% paraformaldehyde, embedded in paraffin, and cut into 4-μm thick sections. H&E staining was performed to observe the histopathological features. After dewaxing and hydration, tissue sections were permeabilized with 0.5% Triton X-100 at room temperature for 10 min, and were then blocked with 3% H_2_O_2_ for 30 min and subsequently with 10% goat serum for 30 min. Next, samples were incubated with antibodies against PHGDH (1:1000, Proteintech) and Ki67 (1:200, Abclonal) at 4°C overnight. Then, sections were stained using the GTVision III Detection System (Gene Tech, China).

### NADPH and GSH Determination

Cells were seeded into 6-well plates, and cultured for 24 h. NADPH and GSH levels were determined using a NADPH Assay Kit (Beyotime) and GSH and GSSG Assay Kit (Beyotime), respectively, according to the manufacturer’s instructions.

### Serum Starvation and Serine Deprivation Treatment

HCT8 and DLD1 cells were seeded into 12-well plates, and cultured in normal medium or serum-free medium for the indicated times. For serine deprivation, cells were cultured in normal medium or serine-free medium for the indicated times. All samples were then prepared for qRT-PCR and western blotting.

### Bioinformatics and Statistical Analyses

Differential gene expression was determined using the edge R package. Potential target genes of circ_62682 were predicted by the Circbank database (http://www.circbank.cn/) and CircInteractome database (https://circinteractome.irp.nia.nih.gov/). Target genes of miR-940 were predicted using TargetScan (http://www.targetscan.org) and miRDB (http://mirdb.org/). Gene enrichment analysis was performed using the clusterProfiler package and Database for Annotation, Visualization and Integrated Discovery (DAVID v6.8, https://david.ncifcrf.gov/). Survival curves and best cut-off point were plotted and determined using the survminer package in R. The detailed patient information is described in Table 1. Data are expressed as the mean ± standard deviation (SD) and processed with GraphPad (San Diego, United States). One-way analysis of variance (ANOVA) and Tukey’s multiple comparison test, or Student’s t test, were used to assess the statistical significance between different groups. A *p* value < 0.05 was considered statistically significant.

**TABLE 1:** Correlation of circ_0062682 expression with clinicopathological features in CRC.

Characteristics	circ_0062682		*p* value
	Low	High	
Age (years)			
﹤60	17	17	0.999
≥ 60	22	20	
Gender			
male	15	22	0.108
female	24	15	
Tumor size (cm)			
﹤5	22	24	0.489
≥ 5	17	13	
Location			
Colon	16	17	0.817
Rectum	23	20	
Differentiation			
Well and moderately	29	27	0.999
Poorly	10	10	
Depth of tumor			
T1+T2	11	11	0.206
T3	19	14	
T4	9	12	
Tumor stage			
Ⅰ+Ⅱ	32	22	0.024^*^
Ⅲ	7	14	
Ⅳ	0	1	

## Results

### Circ_0062682 Is Upregulated in CRC Tissues and Correlated With Poor Prognosis

**FIGURE 1:**
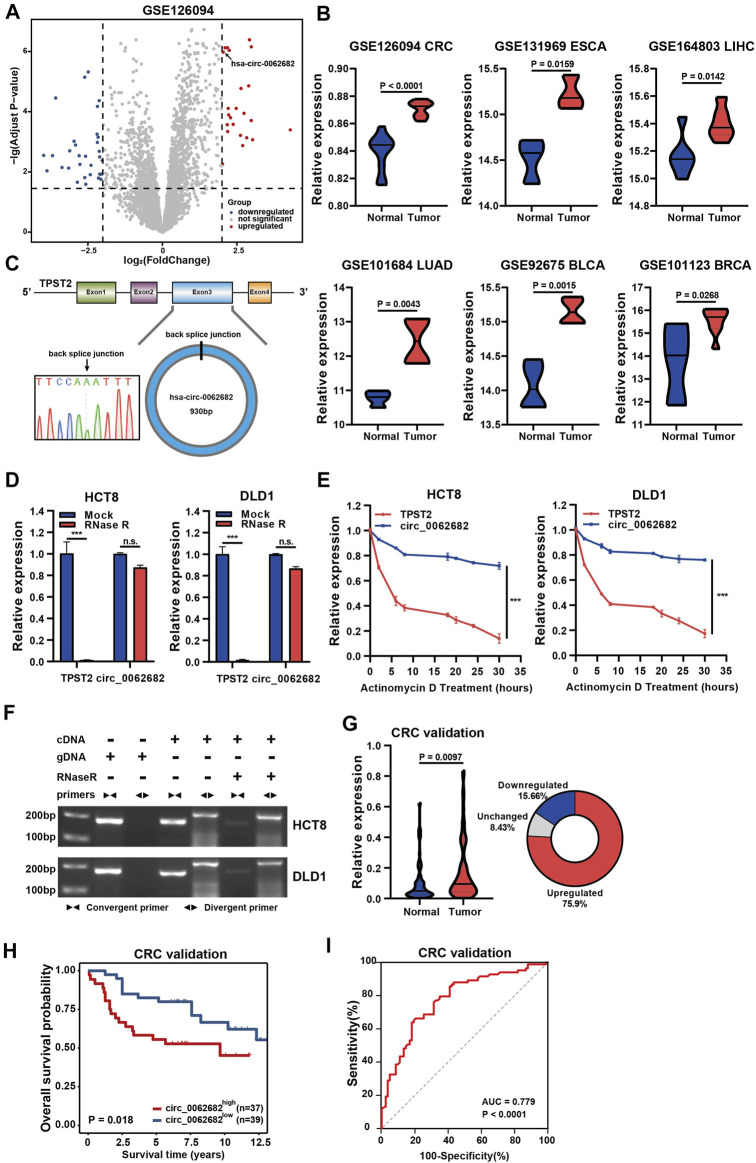
Circ_0062682 is upregulated in CRC and correlated with poor prognosis. **(A)** Volcano plots of differentially expressed circRNAs in CRC vs normal samples. The red points indicate the statistically upregulated circRNAs, the blue points indicate downregulated circRNAs, and the gray points indicate no significant circRNAs. **(B)** Circ_0062682 is upregulated in multiple cancer types based on the analyses of multiple GEO datasets. **(C)** The back splicing site of circ_0062682 was confirmed by Sanger sequencing. **(D,E)** The abundances of circ_0062682 and TPST2 were determined using qRT-PCR in HCT8 and DLD1 cells treated with RNase R **(D)** and actinomycin D**(E)**. **(F)** The existence of circ_0062682 in CRC cells was validated by RT-PCR with convergent and divergent primers. **(G,H)** Circ_0062682 was significantly upregulated in CRC tissues **(G)** and correlated with poor prognosis **(H)**. **(I)** The expression of circ_0062682 differentiated CRC tissues from paired normal tissues using a ROC method. *p < 0.05; **p < 0.001; ***p < 0.001.

A circRNA expression matrix of CRC (GSE126094) was obtained from the GEO database. We used the edge R package to screen the aberrantly expressed circRNAs (|log2FC| > 2, adjusted *p* value < 0.05), and the top 20 abnormally expressed circRNAs are listed in Figure 1A. One of the most upregulated circRNAs with unknown functions, circ_0062682, was selected for further study. Based on analyses in multiple GEO datasets (GSE131969, GSE164803, GSE101684, GSE92675 and GSE101123), we revealed that circ_0062682 is obviously overexpressed in a variety of human cancer types, including CRC, breast cancer, lung cancer, oesophageal carcinoma, and bladder cancer (Figure 1B).

According to the Circbase database (http://www.circbase.org/), circ_0062682 originates from exon 3 of TPST2 and has a length of 930 bp (Figure 1C). We designed specific divergent primers targeting the back spliced junction of circ_0062682 to examine its existence. Sanger sequencing of PCR products confirmed the existence of the back spliced junction (Figure 1C). RNase R and actinomycin D were used to further verify the circular features of circ_0062682, and the results showed that circ_0062682 was much more stable than its linear host gene TPST2 (Figures 1D,E). In addition, we also used convergent primers and divergent primers to amplify TPST2 mRNA and circ_0062682 using cDNA and gDNA from CRC cells, respectively. Gel electrophoresis results showed that circ_0062682 was only detectable in cDNA samples (Figure 1F). Together, these data confirm that circ_0062682 is a circRNA.

To further validate the clinical role of circ_0062682 in CRC. We measured its expression in 83 paired CRC tissues. Circ_0062682 was significantly upregulated in CRC and correlated with poor prognosis (Figures 1G,H). Receiver operating characteristic (ROC) curve and correlation analyses showed that circ_0062682 expression could differentiate CRC tissues from normal tissues (Figure 1I and Table 1), suggesting its potential as a biomarker for CRC.

### Circ_0062682 Enhances CRC Cell Proliferation

**FIGURE 2:**
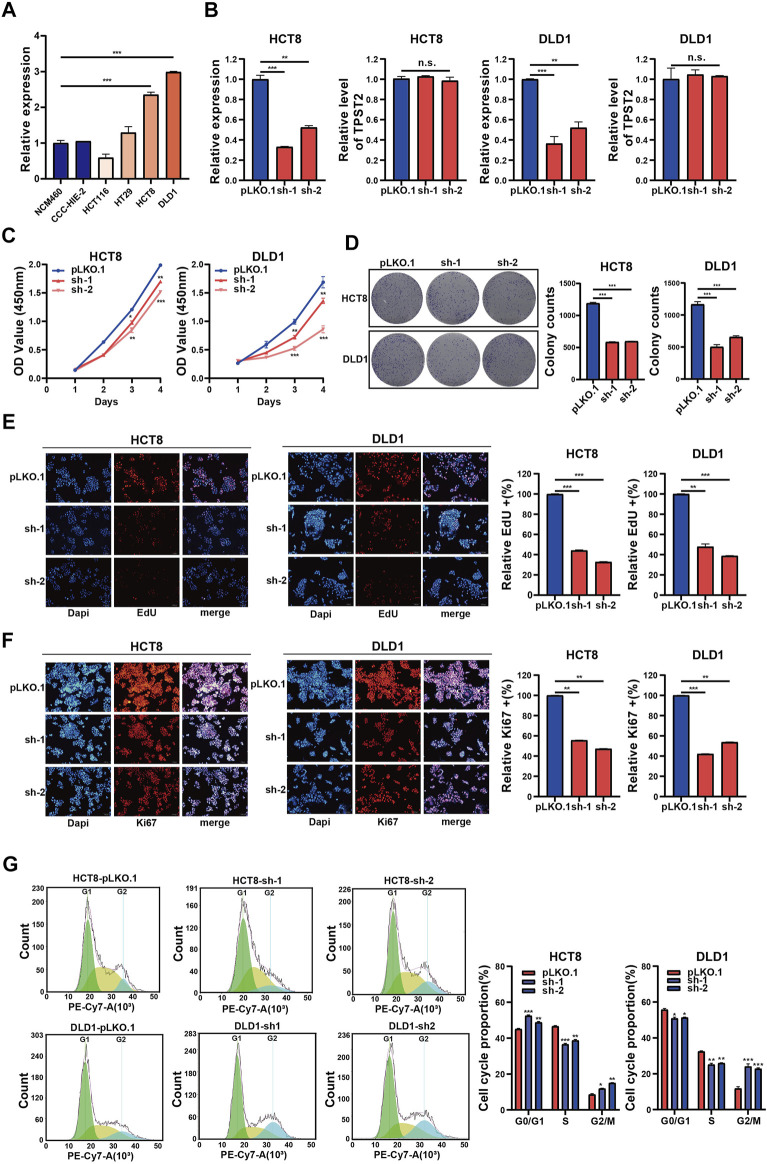
Circ_0062682 knockdown inhibits CRC cell proliferation and cell cycle progression *in vitro*. **(A)** The relative expression of circ_0062682 in different cell lines was detected by qRT-PCR. **(B)** The inhibitory efficiencies of two shRNAs on the expression of circ_0062682 and its host gene TPST2 were evaluated by qRT-PCR in CRC cells. **(C,D)** CCK-8 and colony formation assays were performed to evaluate the effects of circ_0062682 knockdown on the proliferation of CRC cells. **(E,F)** The effects of circ_0062682 knockdown on CRC cell proliferation were assessed by EdU**(E)** and Ki67 staining **(F)**. **(G)** The effects of circ_0062682 knockdown on the cell cycle progression of CRC cells. *p < 0.05; **p < 0.001; ***p < 0.001.

To investigate the functions of circ_0062682 in CRC, we first constructed two circ_0062682-depleted CRC stable cell lines using HCT8 and DLD1 cells that express relatively high endogenous circ_0062682 (Figure 2A). In these two stable cell lines, the expression levels of circ_0062682 were significantly decreased compared with their corresponding control cells, whereas TPST2 expression was unchanged, indicating the specificity of shRNAs (Figure 2B). CCK-8 and colony formation assays indicated that knockdown of circ_0062682 notably reduced the proliferation and colony formation abilities of CRC cells (Figures 2C,D). EdU assay and Ki67 immunofluorescence analyses further confirmed the growth-promoting effects of circ_0062682 in CRC cells (Figures 2E,F). In addition, we also checked the effects of circ_0062682 knockdown on the cell cycle of CRC cells. The results showed that circ_0062682 knockdown resulted in significantly decreased ratios of S-phase and G2/M phase arrest (Figure 2G). Taken together, these data demonstrate that circ_0062682 could promote CRC cell proliferation.

### Circ_0062682 Functions as a Sponge of miR-940

**FIGURE 3:**
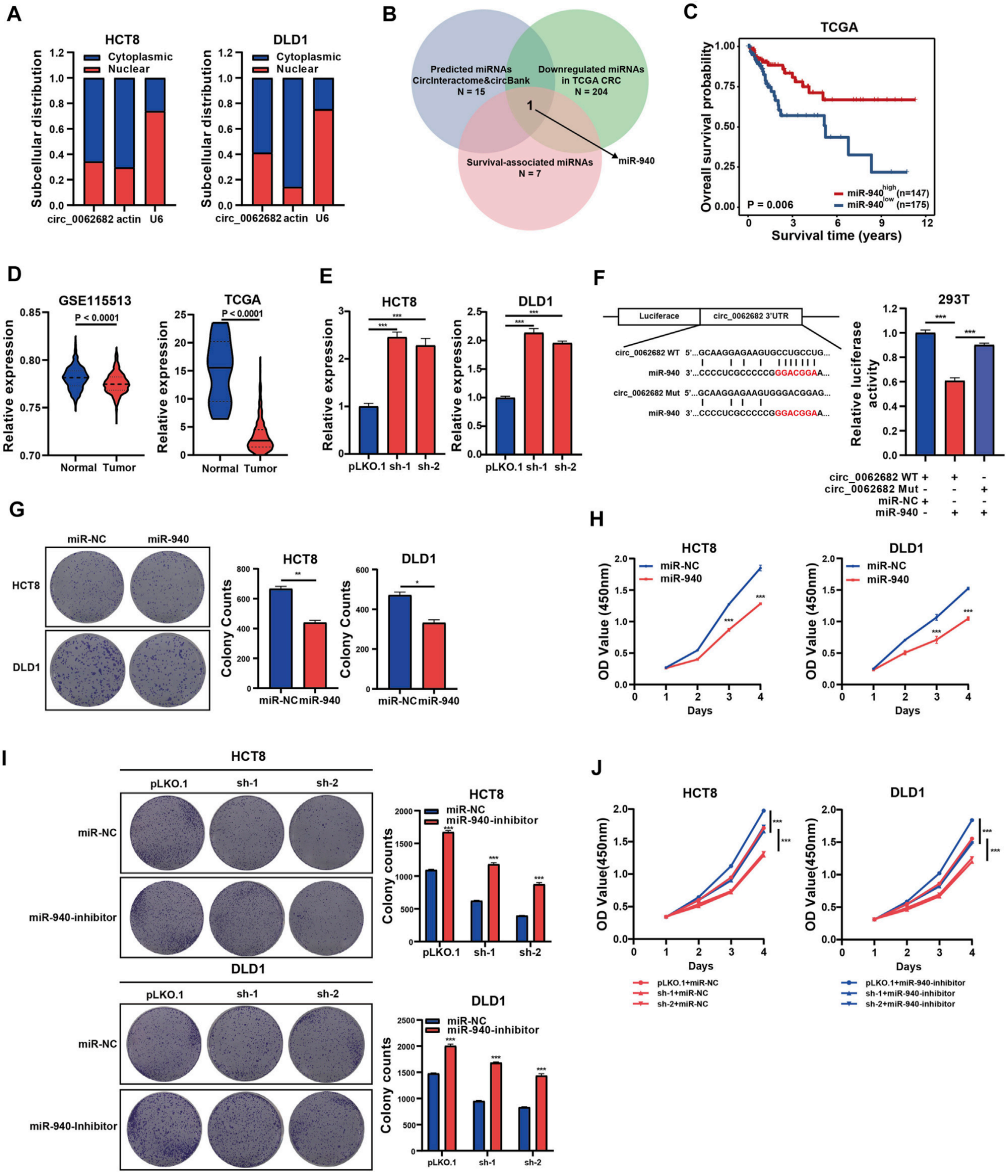
Circ_0062682 functions as a miR-940 sponge. **(A)** Subcellular localization of circ_0062682 in CRC cells. Nuclear and cytoplasmic fractions were subjected to RNA extraction and qRT-PCR. **(B)** Venn diagram showing the overlapping of the target miRNAs of circ_0062682 predicted by CircInteractome and CircBank and by the expression and survival analyses of the TCGA CRC cohort. **(C)** Kaplan-Meier survival analysis of CRC patients with relatively high and low expression of miR-940. The cut-off value for grouping was determined by a ROC method. **(D)** MiR-940 expression in the GSE115513 and TCGA CRC datasets. **(E)** The expression of miR-940 was measured in circ_00626820-silenced CRC cells by qRT-PCR. **(F)** The relative luciferase activities of circ_0062682-WT or circ_0062682-Mut cotransfected with miR-940 mimic were determined by dual luciferase reporter assays. **(G,H)** Ectopic miR-940 expression inhibited the colony formation**(G)** and proliferation **(H)** of CRC cells. **(I,J)** MiR-940 inhibitor rescued the decreased activities of colony formation**(I)** and cell proliferation **(J)** induced by circ_0062682 knockdown in CRC cells. *p < 0.05; **p < 0.001; ***p < 0.001.

Previous studies revealed that circRNAs could function as miRNA sponges to regulate gene expression. Subcellular location analyses revealed that circ_0062682 was mainly enriched in cytoplasm (Figure 3A), and thus we speculated that it might act as a ceRNA in CRC. Therefore, we predicted circ_0062682-associated miRNAs through the CircInteractome and Circbank databases, and screened unearthed survival-related miRNAs with decreased expression in tumor tissues using the TCGA CRC dataset. Using these selection strategies, miR-940 was identified as the only miRNA candidate of circ_0062682 in CRC (Figure 3B). Low levels of miR-940 were associated with poor prognosis in CRC (Figure 3C). We also confirmed that miR-940 was indeed downregulated in CRC using the data from the TCGA and GSE115513 CRC cohorts (Figure 3D). Moreover, the expression levels of miR-940 were obviously decreased in circ_0062682-silenced CRC cells (Figure 3E). To prove the binding between circ_0062682 and miR-940, luciferase reporter vectors of circ_0062682 carrying wild type (WT) or mutated (Mut) miR-940 binding sites were constructed for luciferase reporter assays. The results revealed that miR-940 could inhibit the luciferase activity of the circ_0062682 WT group but not the Mut group, suggesting that circ_0062682 could sponge miR-940 (Figure 3F, Supplementary Figure S1A). Colony formation and CCK-8 assays illustrated that ectopic expression of miR-940 inhibited CRC cell proliferation (Figures 3G,H), whereas inhibition of miR-940 could rescue the proliferation-suppressive effects of circ_0062682 knockdown in CRC cells (Figures 3I,J). Collectively, these data suggest that circ_0062682 could exert its proliferation-inhibitory functions in CRC by sponging miR-940.

### PHGDH is a Novel Target of miR-940 and Mediates the Effects of circ_0062682/miR-940 Signaling on CRC Cell Proliferation

**FIGURE 4:**
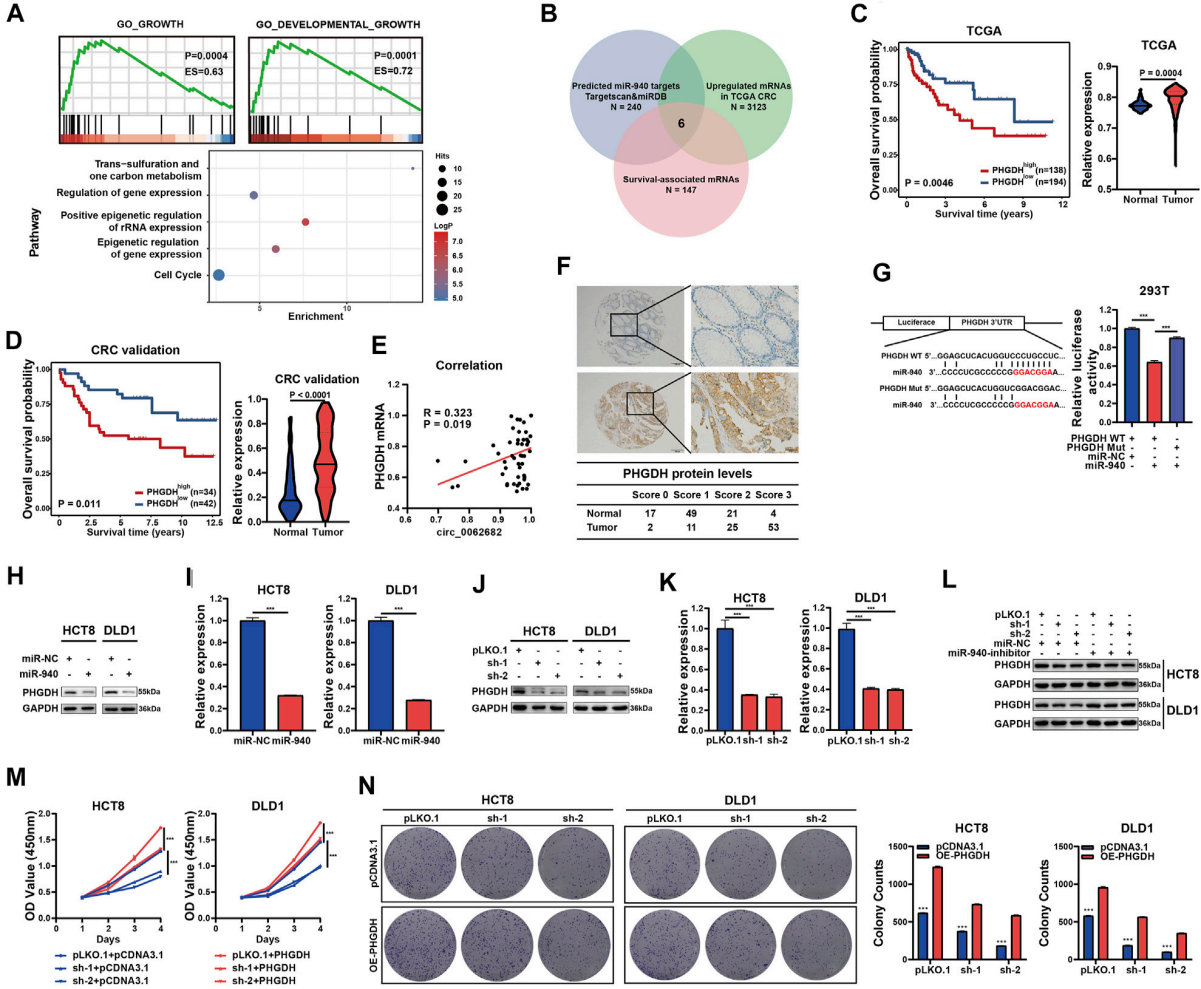
Circ_0062682 upregulates the expression of PHGDH in CRC cells by sponging miR-940. **(A)** Predicted miR-940 target genes were enriched in one carbon metabolism, growth and development-related pathways using GSEA, GO and KEGG enrichment analyses. **(B)** Venn diagram showing the screening results of potential target mRNAs of miR-940. **(C,D)** The significantly upregulated mRNA expression of PHGDH in CRC tissues compared with normal tissues, which correlated with poor prognosis based on the TCGA CRC dataset**(C)** and an independent validation CRC cohort**(D)**. **(E)** A positive correlation between circ_0062682 and PHGDH expression in CRC tissues. **(F)** The protein expression of PHGDH in CRC tissues and NCTs was detected by immunohistochemistry staining. **(G)** The relative luciferase activities of PHGDH-WT or PHGDH-Mut after cotransfection with miR-940 mimic were determined by dual luciferase reporter assays. **(H,I)** The relative protein **(H)** and mRNA expression levels **(I)** of PHGDH were determined by qRT-PCR and western blotting in miR-940-transfected CRC cells, respectively. **(J,K)** The relative protein **(J)** and mRNA**(K)** expression levels of PHGDH were determined in circ_0062682-silenced CRC cells by qRT-PCR and western blotting, respectively. **(L)** MiR-940 inhibitor rescued the decreased protein expression of PHGDH induced by circ_0062682 knockdown in CRC cells. **(M,N)** PHGDH overexpression rescued the decreased activities of cell proliferation **(M)** and colony formation**(N)** induced by circ_0062682 knockdown in CRC cells. *p < 0.05; **p < 0.001; ***p < 0.001.

To investigate potential pathways regulated by miR-940, the target genes of miR-940 were predicted using TargetScan and subjected to GSEA, GO and KEGG enrichment analyses. The results showed that these target genes were mostly enriched in the one carbon metabolism and growth-related signaling pathways (Figure 4A). Among the 240 candidate genes predicted by both TargetScan and miRDB, only six genes (IGFBP1, PHGDH, PTGS2, SLC9A5, VAM21, and ZNF850) were significantly upregulated in CRC and associated with patient survival based on the CRC dataset of TCGA. Of these six genes, PHGDH, a key regulator of one carbon metabolism, was selected for further studies (Figure 4B). PHGDH mRNA levels were obviously increased in CRC and correlated with poor survival in the CRC cohort of TCGA (Figure 4C). Similarly, we confirmed the overexpression of PHGDH using qRT-PCR in an independent CRC validation cohort (Figure 4D) and observed a positive correlation between circ_0062682 expression and PHGDH mRNA levels (Figure 4E). In addition, we further detected the protein expression of PHGDH using IHC and observed increased protein levels of PHGDP in CRC tissues compared with NCTs (Figure 4F).

To determine whether PHGDH is a target of miR-940, we constructed luciferase reporter vectors of the PHGDH 3’UTR with WT or Mut miR-940 binding sites (Figure 4G). Luciferase assay results revealed the regulation of PHGDH by miR-940 (Figure 4G, Supplementary Figure S1B). In addition, both ectopic miR-940 expression and circ_0062682 knockdown could decrease PHGDH expression in CRC cells (Figures 4H–K). Moreover, the effects of circ_0062682 knockdown on PHGDH expression and CRC cell proliferation could be rescued by the miR-940 inhibitor and ectopic expression of PHGDH (Figures 4L–N). Together, these results suggest that circ_0062682 promotes CRC proliferation by regulating miR-940/PHGDH signaling.

### Circ_0062682 Promotes Serine Metabolism Through the miR-940/PHGDH Axis

**FIGURE 5:**
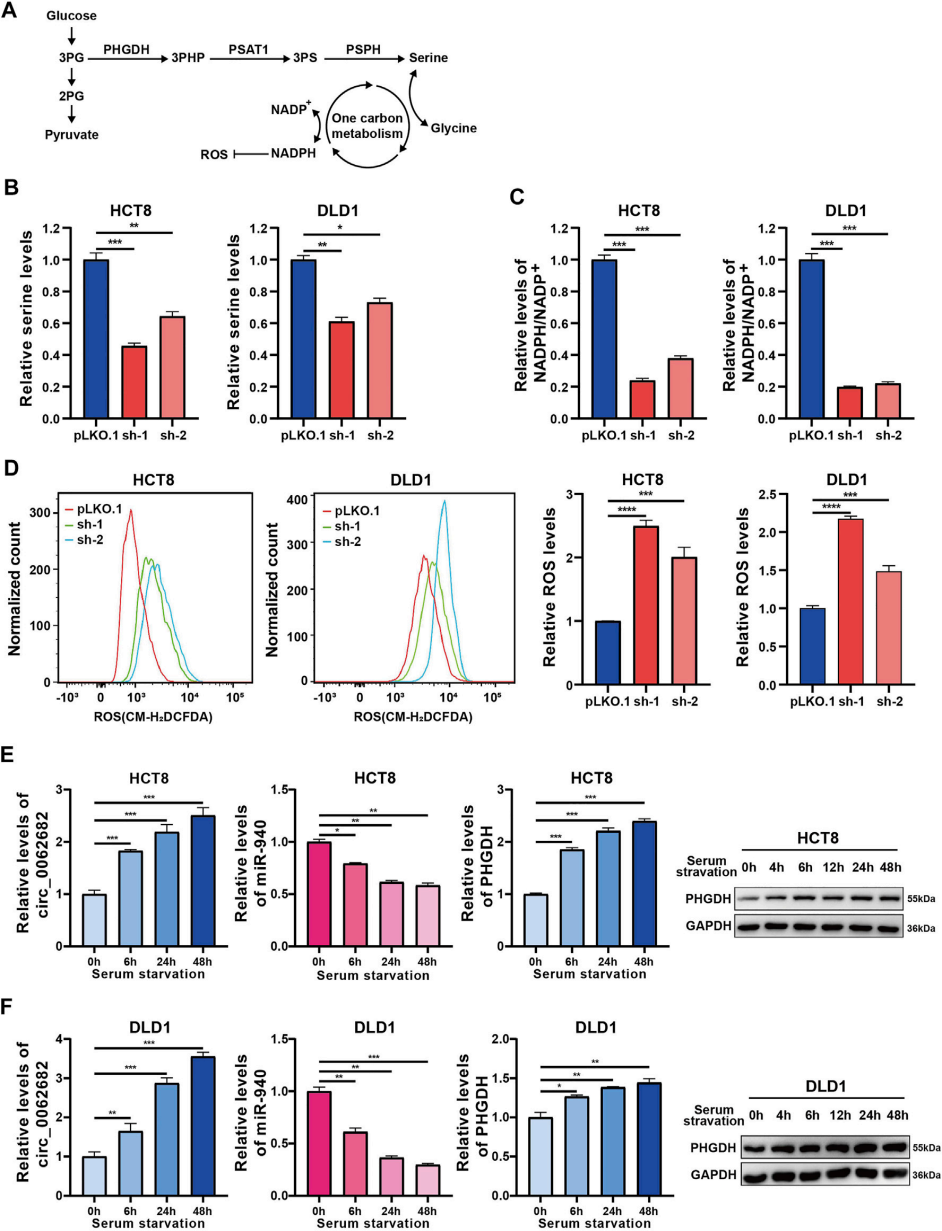
Circ_0062682 promotes serine metabolism through the miR-940/PHGDH axis. **(A)** De novo serine biosynthesis diverges from glycolysis. **(B)** Intracellular serine levels were measured by LC-MS in circ_0062682-silenced CRC cells. **(C)** Intracellular NADPH levels were measured in circ_0062682-silenced CRC cells. **(D)** ROS levels were measured in circ_0062682-silenced CRC cells. **(E,F)** The expression of circ_0062682, miR-940, and PHGDH in CRC cells was measured after serum starvation for the indicated times by qRT-PCR or western blotting. *p < 0.05; **p < 0.001; ***p < 0.001.

As the rate-limiting enzyme of the serine biosynthesis pathway (Figure 5A), PHGDH is overexpressed in various types of cancer, and appears to be a promising target for cancer therapy. We first evaluated the regulatory effects of circ_0062682 on serine metabolism, and revealed that circ_0062682 knockdown could reduce the serine and glycine levels in CRC cells (Figure 5B, Supplementary Figures S1C–D). Secondly, we demonstrated that silencing circ_0062682 expression also scaled down NADPH/NADP^+^ and GSH levels and induced ROS accumulation in CRC cells (Figures 5C,D, Supplementary Figures S1E,F). To survive under nutrient stress conditions, cancer cells could increase serine biosynthesis. To determine why circ_0062682 expression is enhanced in CRC, we measured the effects of serum starvation and oxidative stress on the expression of circ_0062682. Eventually, we discovered that the expression of circ_0062682 and PHGDH was increased, whereas miR-940 was downregulated in CRC cells under serum starvation (Figures 5E,F). Considering that PHGDH plays a vital role in serine metabolism, we also found that serine deprivation could induce the expression of circ_0062682, thus facilitating PHGDH expression (Supplementary Figures S1G,H). These results suggest that circ_0062682 promotes serine metabolism through the miR-940/PHGDH axis.

### Circ_0062682 Knockdown Inhibits Tumor Growth *in vivo*

**FIGURE 6:**
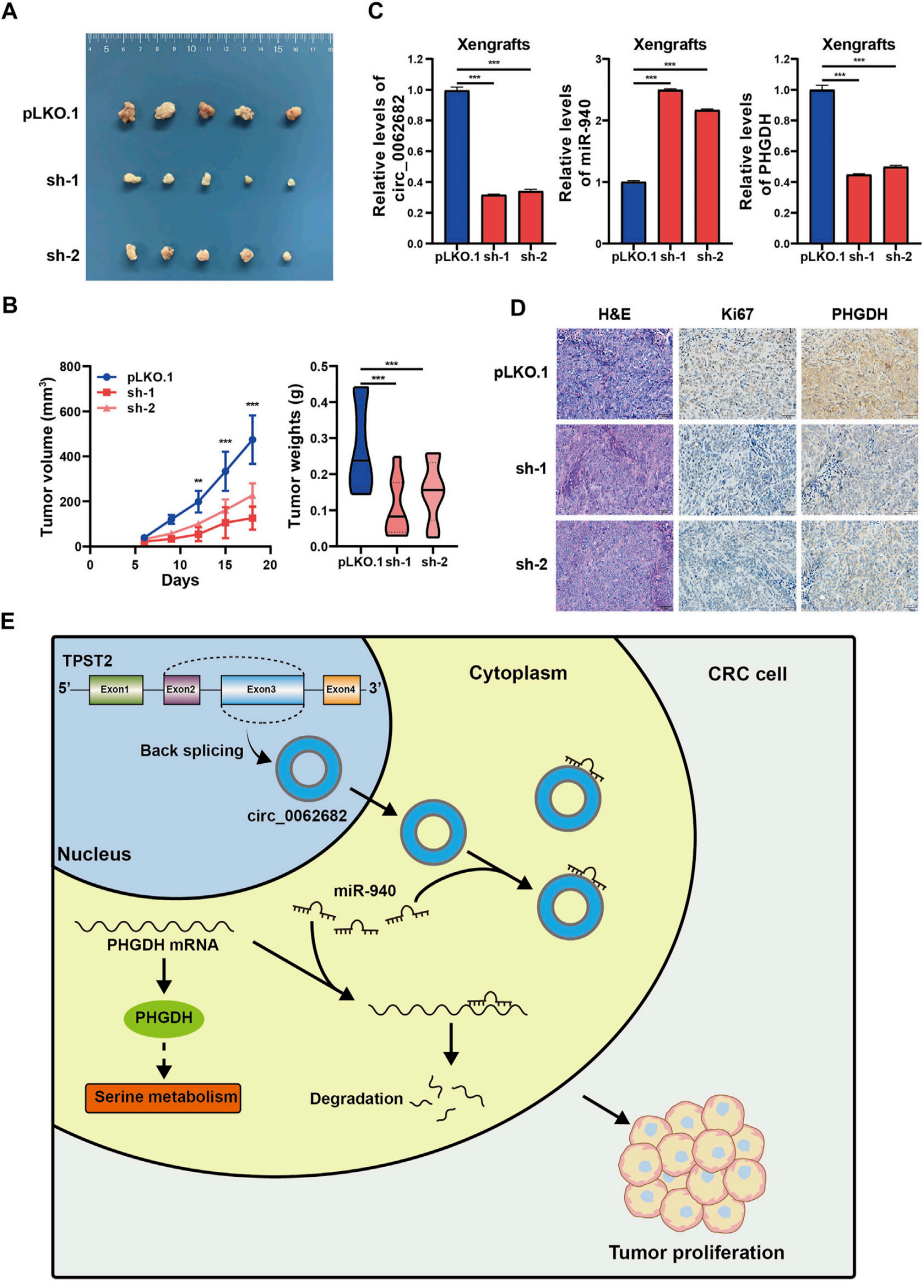
Circ_0062682 knockdown inhibits CRC growth *in vivo*. **(A,B)** DLD1 cells with stable knockdown of circ_0062682 were injected subcutaneously into nude mice (*n* = 5) to evaluate the effect of circ_0062682 knockdown on tumor formation **(A)**. The tumor volumes were measured every 3 days and the tumor weights were recorded after mouse sacrifice **(B)**. **(C)** The expression levels of circ_0062682, miR-940, and PHGDH mRNA were measured in resected xenografts from **(A)** by qRT-PCR. **(D)** The protein expression of Ki67 and PHGDH in xenografts was detected by IHC staining. **(E)** A mechanistic model depicting the growth-regulatory role of the circ_0062682/miR-940/PHGDH axis in CRC.*p < 0.05; **p < 0.001; ***p < 0.001.

To further study the growth-regulatory function of circ_0062682 *in vivo*, a subcuntaneous tumor xenograft model was constructed using DLD1 cells with stable knockdown of circ_0062682. The results showed that silencing circ_0062682 expression obviously slowed tumor growth (Figures 6A,B). We examined the expression levels of circ_0062682, miR-940 and PHGDH, and confirmed that circ_0062682 knockdown increased miR-940 expression and decreased PHGDH levels in these xenografts (Figure 6C). In addition, IHC staining indicated that circ_0062682 knockdown reduced the expression of PHGDH and Ki67 in these tumor tissues (Figure 6D). Together, these data confirm that circ_0062682 knockdown inhibits CRC tumor growth *in vivo* via the miR-940/PHGDH axis (Figure 6E).

## Discussion

In the anabolic pathway, the serine biosynthesis pathway is the key turning point of glucose transformation. Imported serine and serine from the glycolytic branch can be converted to glycine, which is further utilized to promote nucleotide synthesis and GSH production. Despite these significant advances, the detailed mechanisms by which cancer cells regulate serine metabolism under stressed conditions remain largely elusive. In this study, we reported for the first time that circ_0062682, as a stress-induced circRNA, is able to promote serine synthesis and tumor growth by regulating the miR-940/PHGDH axis. Our study, therefore, demonstrates a previously unappreciated function of circRNA in supporting cancer cell survival under stressed conditions.

Growing evidence has uncovered that circRNAs are frequently deregulated and play vital roles in human cancers (Fontemaggi et al., 2021), including CRC (Li et al., 2018; Li Y. et al., 2021; Yu and Lei, 2021). Several studies have revealed the regulatory roles of circRNAs in cellular metabolism (He et al., 2017; Li et al., 2019c; Yu et al., 2019). For example, circACC1, produced from ACC1 in response to serum deprivation, modulates both fatty acid β-oxidation and glycolysis, leading to significant changes in cellular lipid storage (Li et al., 2019c). Xing et al. reported that circPDCD11 enhances aerobic glycolysis and tumor progression by regulating miR-432-5p/LDHA signaling in triple-negative breast cancer (Xing et al., 2021). Li and colleagues revealed that circMAT2B promotes glycolysis and tumor progression by regulating the circMAT2B/miR-338-3p/PKM2 axis under hypoxia conditions (Li et al., 2019b). However, to date, the roles of circRNAs in cancer metabolism reprogramming have not been fully characterized.

In the present study, we identified a functionally unknown circRNA,circ_0062682, with aberrant overexpression across cancers, including CRC. Circ_0062682 is a circular transcript of its host gene TPST2 with a length of 930 bp. We observed significantly increased expression of circ_0062682 in CRC, which was associated with poor survival based on data from multiple CRC cohorts. Functionally, circ_0062682 could promote CRC cell proliferation both *in vitro* and *in vivo*, suggesting that circ_0062682 functions as an oncogene in CRC. In view of the extensive upregulation of circ_0062682 in multiple cancer types, it may play a key role in human cancers and is a potential pan-cancer therapeutic target.

A key mechanism of cytoplasmic circRNA is to act as ceRNAs to regulate miRNA functions. Circ_0062682 was predominantly localized in the cytoplasm, suggesting that it might exert functions through a ceRNA mechanism. By a series of bioinformatic and experimental validations, we revealed that circ_0062682 executed its tumor-promoting function by sponging miR-940. Although previous studies reported that miR-940 could function as an oncogene or a tumor suppressor in different cancer types (Li H. et al., 2021), a recent study reported that miR-940 played a tumor-inhibitory role in CRC (Wang et al., 2019). Based on the data from multiple CRC cohorts, we further confirmed the downregulation of miR-940 in CRC tissues, which was associated with poor clinical outcomes. Functional assays also demonstrated that miR-940 inhibited CRC cell proliferation, confirming the tumor-suppressive role of miR-940 in CRC.

Activation of the serine synthesis pathway is very important for the proliferation and survival of cancer cells under nutrient stress conditions, through which the glycolytic intermediate 3-PG is transformed into serine (Sun et al., 2015). Serine plays a key role in the biosynthesis of many molecules, supporting NADPH and nucleotide production. As an active oxygen scavenger, NADPH is very important to maintain redox balance (Bedard and Krause, 2007). Here, we provide the first evidence that circ_0062682 activates the serine synthesis pathway by regulating the miR-940/PHGDH axis, thereby increasing NADPH and GSH levels and reducing ROS levels in CRC cells to promote the survival of cancer cells under stress conditions. These data demonstrate an important regulatory function of circ_0062682 in the serine synthesis pathway.

PHGDH is frequently overexpressed in multiple human cancers and is associated with poor prognosis in cancer patients. The enhanced expression of PHGDH activates serine synthesis and then promotes cancer growth and progression (Possemato et al., 2011; Jia et al., 2016; Zhang et al., 2017; Wang et al., 2020). Accumulating evidence suggests that PHGDH is a promising therapeutic target for cancer. Indeed, several preclinical trials have proven that the targeted inhibition of PHGDH with small molecule inhibitors disrupts the serine synthesis pathway and restricts tumor growth and metastasis (Mullarky et al., 2016; Pacold et al., 2016; Ngo et al., 2020). Since PHGDH plays a central role in modulating serine biosynthesis, it is reasonable that PHGDH expression should be precisely regulated at different angles. PHGDH was proven to be transcriptionally activated by the transcription factors ATF4 and c-Myc, and epigenetically mediated by lysine methyltransferase G9A (Adams, 2007; Ding et al., 2013; DeNicola et al., 2015). In terms of post-transcriptional modifications, the stability of PHGDH can be regulated by the ubiquitin ligases Parkin and RNF5(Liu et al., 2020; Wang et al., 2020). Moreover, a recent study showed that linc01564 increased the expression levels of PHGDH by sequestering miR-107/103a-3p in hepatocellular carcinoma (Zhang et al., 2021). Here, we demonstrated that PHGDH is a novel functional target of miR-940, and circ_0062682 could bind and inhibit miR-940, restoring the tumor-promoting activity of PHGDH in CRC. These results uncover a novel regulatory circ_0062682/miR-940/PHGDH axis in CRC and highlight an important role of circRNAs in the serine metabolism and tumorigenesis.

In summary, this study demonstrates, for the first time, that circ_0062682, a serum and serine deprivation-induced circRNA, promotes serine metabolism and tumor growth in CRC by regulating the miR-940/PHGDH axis, which not only helps to understand the detailed mechanism of cancer cells adapting to stress states, but also suggests that circ_0062682 is a potential novel therapeutic target for CRC.

## Data Availability

The datasets presented in this study can be found in online repositories. The names of the repository/repositories and accession number(s) can be found in the article/Supplementary Material.

## References

[B1] Adams C. M. (2007). Role of the Transcription Factor ATF4 in the Anabolic Actions of Insulin and the Anti-anabolic Actions of Glucocorticoids. J. Biol. Chem. 282 (23), 16744–16753. 10.1074/jbc.m610510200 17430894

[B2] Amelio I. Cutruzzolá F. Antonov A. Agostini M. Melino G. (2014). Serine and glycine Metabolism in Cancer. Trends Biochem. Sci. 39 (4), 191–198. 10.1016/j.tibs.2014.02.004 24657017 PMC3989988

[B3] Ashwal-Fluss R. Meyer M. Pamudurti N. R. Ivanov A. Bartok O. Hanan M. (2014). circRNA Biogenesis Competes with Pre-mRNA Splicing. Mol. Cel. 56 (1), 55–66. 10.1016/j.molcel.2014.08.019 25242144

[B4] Bedard K. Krause K.-H. (2007). The NOX Family of ROS-Generating NADPH Oxidases: Physiology and Pathophysiology. Physiol. Rev. 87 (1), 245–313. 10.1152/physrev.00044.2005 17237347

[B5] Bian Z. Zhang J. Li M. Feng Y. Wang X. Zhang J. (2018). LncRNA-FEZF1-AS1 Promotes Tumor Proliferation and Metastasis in Colorectal Cancer by Regulating PKM2 Signaling. Clin. Cancer Res. 24 (19), 4808–4819. 10.1158/1078-0432.CCR-17-2967 29914894

[B6] Boroughs L. K. DeBerardinis R. J. (2015). Metabolic Pathways Promoting Cancer Cell Survival and Growth. Nat. Cell Biol 17 (4), 351–359. 10.1038/ncb3124 25774832 PMC4939711

[B7] Chen L.-L. (2020). The Expanding Regulatory Mechanisms and Cellular Functions of Circular RNAs. Nat. Rev. Mol. Cell Biol 21 (8), 475–490. 10.1038/s41580-020-0243-y 32366901

[B8] DeNicola G. M. Chen P.-H. Mullarky E. Sudderth J. A. Hu Z. Wu D. (2015). NRF2 Regulates Serine Biosynthesis in Non-small Cell Lung Cancer. Nat. Genet. 47 (12), 1475–1481. 10.1038/ng.3421 26482881 PMC4721512

[B9] Ding J. Li T. Wang X. Zhao E. Choi J.-H. Yang L. (2013). The Histone H3 Methyltransferase G9A Epigenetically Activates the Serine-glycine Synthesis Pathway to Sustain Cancer Cell Survival and Proliferation. Cell Metab. 18 (6), 896–907. 10.1016/j.cmet.2013.11.004 24315373 PMC3878056

[B10] Fontemaggi G. Turco C. Esposito G. Di Agostino S. (2021). New Molecular Mechanisms and Clinical Impact of circRNAs in Human Cancer. Cancers 13 (13), 3154. 10.3390/cancers13133154 34202482 PMC8268751

[B11] He R. Liu P. Xie X. Zhou Y. Liao Q. Xiong W. (2017). circGFRA1 and GFRA1 Act as ceRNAs in Triple Negative Breast Cancer by Regulating miR-34a. J. Exp. Clin. Cancer Res. 36 (1), 145. 10.1186/s13046-017-0614-1 29037220 PMC5644184

[B12] Jia X.-q. Zhang S. Zhu H.-j. Wang W. Zhu J.-h. Wang X.-d. (2016). Increased Expression of PHGDH and Prognostic Significance in Colorectal Cancer. Translational Oncol. 9 (3), 191–196. 10.1016/j.tranon.2016.03.006 PMC4907894 27267836

[B13] Jin G. Liu Y. Zhang J. Bian Z. Yao S. Fei B. (2019). A Panel of Serum Exosomal microRNAs as Predictive Markers for Chemoresistance in Advanced Colorectal Cancer. Cancer Chemother. Pharmacol. 84 (2), 315–325. 10.1007/s00280-019-03867-6 31089750

[B14] Kristensen L. S. Andersen M. S. Stagsted L. V. W. Ebbesen K. K. Hansen T. B. Kjems J. (2019). The Biogenesis, Biology and Characterization of Circular RNAs. Nat. Rev. Genet. 20 (11), 675–691. 10.1038/s41576-019-0158-7 31395983

[B15] Li A. M. Ye J. (2020). Reprogramming of Serine, glycine and One-Carbon Metabolism in Cancer. Biochim. Biophys. Acta (Bba) - Mol. Basis Dis. 1866 (10), 165841. 10.1016/j.bbadis.2020.165841 PMC7442608 32439610

[B16] Li H. Li Y. Tian D. Zhang J. Duan S. (2021a). miR-940 Is a New Biomarker with Tumor Diagnostic and Prognostic Value. Mol. Ther. - Nucleic Acids 25, 53–66. 10.1016/j.omtn.2021.05.003 34168918 PMC8192490

[B17] Li M. Bian Z. Jin G. Zhang J. Yao S. Feng Y. (2019a). Lnc RNA ‐ SNHG 15 Enhances Cell Proliferation in Colorectal Cancer by Inhibiting miR‐338‐3p. Cancer Med. 8 (5), 2404–2413. 10.1002/cam4.2105 30945457 PMC6536931

[B18] Li Q. Pan X. Zhu D. Deng Z. Jiang R. Wang X. (2019b). Circular RNA MAT2B Promotes Glycolysis and Malignancy of Hepatocellular Carcinoma through the miR‐338‐3p/PKM2 Axis under Hypoxic Stress. Hepatology 70 (4), 1298–1316. 10.1002/hep.30671 31004447

[B19] Li Q. Wang Y. Wu S. Zhou Z. Ding X. Shi R. (2019c). CircACC1 Regulates Assembly and Activation of AMPK Complex under Metabolic Stress. Cell Metab. 30 (1), 157–173. 10.1016/j.cmet.2019.05.009 31155494

[B20] Li X.-N. Wang Z.-J. Ye C.-X. Zhao B.-C. Li Z.-L. Yang Y. (2018). RNA Sequencing Reveals the Expression Profiles of circRNA and Indicates that circDDX17 Acts as a Tumor Suppressor in Colorectal Cancer. J. Exp. Clin. Cancer Res. 37 (1), 325. 10.1186/s13046-018-1006-x 30591054 PMC6307166

[B21] Li Y. Chen B. Zhao J. Li Q. Chen S. Guo T. (2021b). HNRNPL Circularizes ARHGAP35 to Produce an Oncogenic Protein. Adv. Sci. 8 (13), 2001701. 10.1002/advs.202001701 PMC8261482 34258149

[B22] Liu J. Zhang C. Wu H. Sun X.-X. Li Y. Huang S. (2020). Parkin Ubiquitinates Phosphoglycerate Dehydrogenase to Suppress Serine Synthesis and Tumor Progression. J. Clin. Invest. 130 (6), 3253–3269. 10.1172/jci132876 32478681 PMC7260041

[B23] Ma S. Kong S. Wang F. Ju S. (2020). CircRNAs: Biogenesis, Functions, and Role in Drug-Resistant Tumours. Mol. Cancer 19 (1), 119. 10.1186/s12943-020-01231-4 32758239 PMC7409473

[B24] Meng S. Zhou H. Feng Z. Xu Z. Tang Y. Li P. (2017). CircRNA: Functions and Properties of a Novel Potential Biomarker for Cancer. Mol. Cancer 16 (1), 94–98. 10.1186/s12943-017-0663-2 28535767 PMC5440908

[B25] Mullarky E. Lucki N. C. Beheshti Zavareh R. Anglin J. L. Gomes A. P. Nicolay B. N. (2016). Identification of a Small Molecule Inhibitor of 3-phosphoglycerate Dehydrogenase to Target Serine Biosynthesis in Cancers. Proc. Natl. Acad. Sci. USA 113 (7), 1778–1783. 10.1073/pnas.1521548113 26831078 PMC4763784

[B26] Navarro M. Nicolas A. Ferrandez A. Lanas A. (2017). Colorectal Cancer Population Screening Programs Worldwide in 2016: An Update. Wjg 23 (20), 3632–3642. 10.3748/wjg.v23.i20.3632 28611516 PMC5449420

[B27] Ngo B. Kim E. Osorio-Vasquez V. Doll S. Bustraan S. Liang R. J. (2020). Limited Environmental Serine and Glycine Confer Brain Metastasis Sensitivity to PHGDH Inhibition. Cancer Discov. 10 (9), 1352–1373. 10.1158/2159-8290.CD-19-1228 32571778 PMC7483776

[B28] Pacold M. E. Brimacombe K. R. Chan S. H. Rohde J. M. Lewis C. A. Swier L. J. Y. M. (2016). A PHGDH Inhibitor Reveals Coordination of Serine Synthesis and One-Carbon Unit Fate. Nat. Chem. Biol. 12 (6), 452–458. 10.1038/nchembio.2070 27110680 PMC4871733

[B29] Pan S. Fan M. Liu Z. Li X. Wang H. (2021). Serine, glycine and One-carbon M-etabolism in C-ancer (Review). Int. J. Oncol. 58 (2), 158–170. 10.3892/ijo.2020.5158 33491748 PMC7864012

[B30] Patop I. L. Wüst S. Kadener S. (2019). Past, Present, and Future of circRNAs. EMBO J. 38 (16), e100836. 10.15252/embj.2018100836 31343080 PMC6694216

[B31] Possemato R. Marks K. M. Shaul Y. D. Pacold M. E. Kim D. Birsoy K. (2011). Functional Genomics Reveal that the Serine Synthesis Pathway Is Essential in Breast Cancer. Nature 476 (7360), 346–350. 10.1038/nature10350 21760589 PMC3353325

[B32] Sun L. Song L. Wan Q. Wu G. Li X. Wang Y. (2015). cMyc-Mediated Activation of Serine Biosynthesis Pathway Is Critical for Cancer Progression under Nutrient Deprivation Conditions. Cell Res 25 (4), 429–444. 10.1038/cr.2015.33 25793315 PMC4387561

[B33] Sung H. Ferlay J. Siegel R. L. Laversanne M. Soerjomataram I. Jemal A. (2021). Global Cancer Statistics 2020: GLOBOCAN Estimates of Incidence and Mortality Worldwide for 36 Cancers in 185 Countries. CA A. Cancer J. Clin. 71 (3), 209–249. 10.3322/caac.21660 33538338

[B34] Wang C. Wan X. Yu T. Huang Z. Shen C. Qi Q. (2020). Acetylation Stabilizes Phosphoglycerate Dehydrogenase by Disrupting the Interaction of E3 Ligase RNF5 to Promote Breast Tumorigenesis. Cell Rep. 32 (6), 108021. 10.1016/j.celrep.2020.108021 32783943

[B35] Wang Y. Zhao M. Zhao H. Cheng S. Bai R. Song M. (2019). MicroRNA-940 Restricts the Expression of Metastasis-Associated Gene MACC1 and Enhances the Antitumor Effect of Anlotinib on Colorectal Cancer. Ott 12, 2809–2822. 10.2147/ott.s195364 PMC6489584 31114229

[B36] Xing Z. Wang R. Wang X. Liu J. Zhang M. Feng K. (2021). CircRNA Circ-PDCD11 Promotes Triple-Negative Breast Cancer Progression via Enhancing Aerobic Glycolysis. Cell Death Discov. 7 (1), 218. 10.1038/s41420-021-00604-y 34420029 PMC8380247

[B37] Yin Y. Long J. He Q. Li Y. Liao Y. He P. (2019). Emerging Roles of circRNA in Formation and Progression of Cancer. J. Cancer 10 (21), 5015–5021. 10.7150/jca.30828 31602252 PMC6775606

[B38] Yu T. Wang Y. Fan Y. Fang N. Wang T. Xu T. (2019). CircRNAs in Cancer Metabolism: a Review. J. Hematol. Oncol. 12 (1), 90. 10.1186/s13045-019-0776-8 31484561 PMC6727394

[B39] Yu Y. Lei X. (2021). CircFAM120B Blocks the Development of Colorectal Cancer by Activating TGF-Beta Receptor II Expression via Targeting miR-645. Front. Cell Dev. Biol. 9, 682543. 10.3389/fcell.2021.682543 34381772 PMC8350741

[B40] Zhang B. Zheng A. Hydbring P. Ambroise G. Ouchida A. T. Goiny M. (2017). PHGDH Defines a Metabolic Subtype in Lung Adenocarcinomas with Poor Prognosis. Cell Rep. 19 (11), 2289–2303. 10.1016/j.celrep.2017.05.067 28614715

[B41] Zhang G. Yang Y. Hu H. Liu K. Li B. Zhu Y. (2021). Energy Stress-Induced Linc01564 Activates the Serine Synthesis Pathway and Facilitates Hepatocellular Carcinogenesis. Oncogene 40 (16), 2936–2951. 10.1038/s41388-021-01749-x 33742121

