# Supplementary material for: Hsa_circ_0062682 Promotes Serine Metabolism and Tumor Growth in Colorectal Cancer by Regulating the miR-940/PHGDH Axis
Source: Front Cell Dev Biol. 2021 Dec 8;9:770006. doi: 10.3389/fcell.2021.770006 (PMC8692793; doi:10.3389/fcell.2021.770006)
Supplement: Supplementary file 2 [file Table1.docx]

|  | **circ62682** | |  |
| --- | --- | --- | --- |
| **Characteristics** | **Low** | **High** | **P value** |
| Age (years) |  |  |  |
| ＜60 | 17 | 20 | 0.865 |
| ≥ 60 | 22 | 24 |  |
| Gender |  |  |  |
| male | 16 | 25 | 0.062 |
| female | 25 | 17 |  |
| Tumor size (cm) |  |  |  |
| ＜5 | 22 | 25 | 0.970 |
| ≥ 5 | 17 | 19 |  |
| Location |  |  |  |
| Colon | 16 | 20 | 0.648 |
| Rectum | 23 | 24 |  |
| Differentiation |  |  |  |
| Well and moderately | 29 | 27 | 0.346 |
| Poorly | 11 | 16 |  |
| Depth of tumor |  |  |  |
| T1+T2 | 11 | 12 | 0.006^*^ |
| T3 | 19 | 16 |  |
| T4 | 9 | 16 |  |
| Tumor stage |  |  |  |
| Ⅰ+Ⅱ | 32 | 23 | <0.001^*^ |
| Ⅲ | 7 | 17 |  |
| Ⅳ | 0 | 4 |  |

**Supplementary table 1: Correlation of circ_0062682 expression with clinicopathological features in CRC for all patients**
