# Supplementary figures and images for: Hsa_circ_0062682 Promotes Serine Metabolism and Tumor Growth in Colorectal Cancer by Regulating the miR-940/PHGDH Axis
Source: Front Cell Dev Biol. 2021 Dec 8;9:770006. doi: 10.3389/fcell.2021.770006 (PMC8692793; doi:10.3389/fcell.2021.770006)

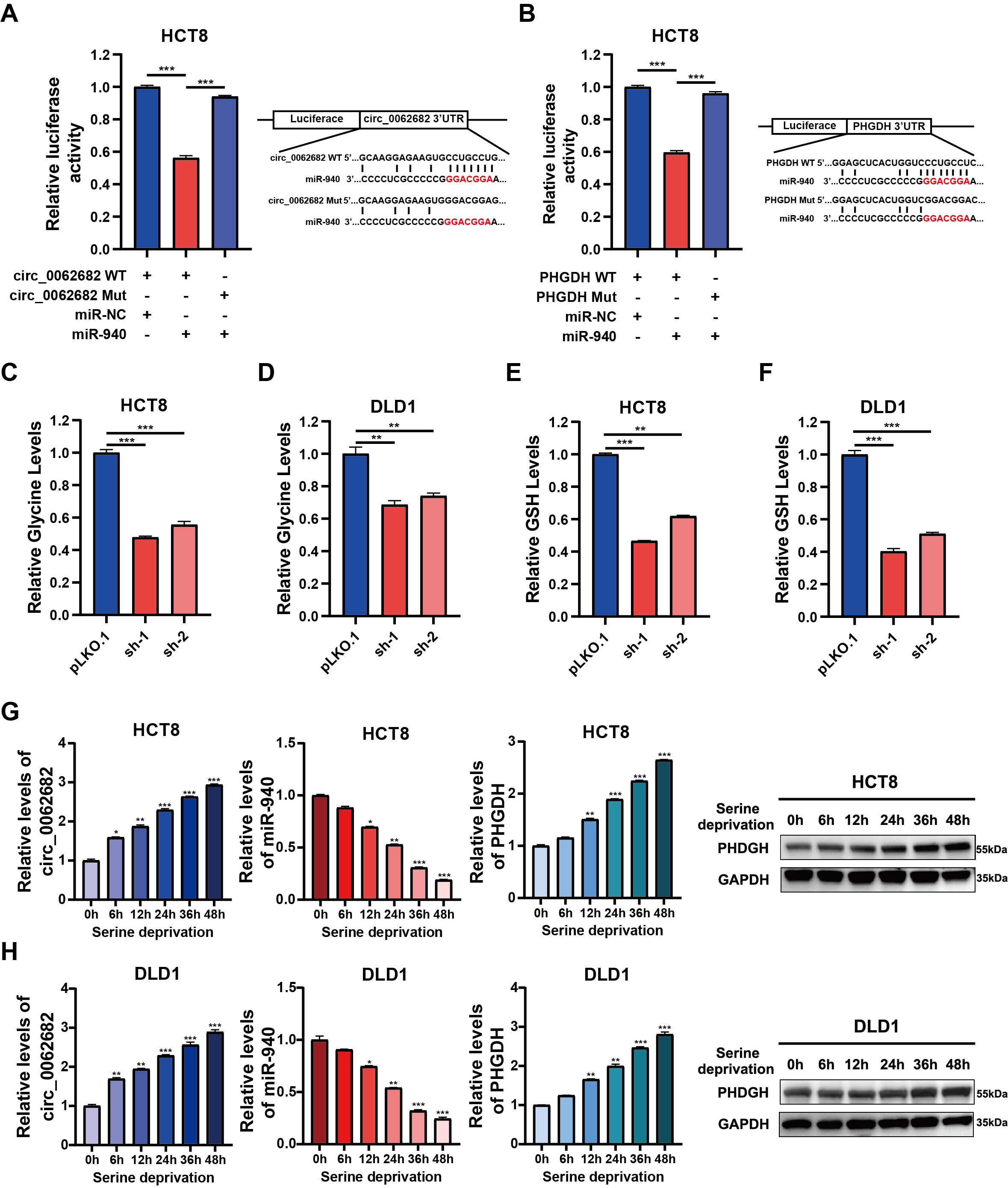

Supplement: Supplementary file 3 [file Image1.JPEG]
